# Evaluation and treatment of low and anxious mood in Chinese-speaking international students studying in Scotland: study protocol of a pilot randomised controlled trial

**DOI:** 10.1186/s40814-015-0019-x

**Published:** 2015-06-09

**Authors:** Mengyi Zheng, Carrie-Anne McClay, Sarah Wilson, Christopher Williams

**Affiliations:** Institute of Health and Wellbeing, University of Glasgow, Gartnavel Royal Hospital, Administration Building, 1055 Great Western Road, Glasgow City, G12 0XH UK

**Keywords:** Depression, Low mood, cCBT, Guided self-help, Online, Psychotherapy, RCT, International students, Treatment gap, Bibliotherapy, Life skills, Living life to the full, Stress, Anxiety, Students, Chinese, Chinese speaking

## Abstract

**Background:**

Low mood is a common mental health problem affecting up to 121 million people worldwide and is common in students, particularly international students. Cognitive behavioural therapy (CBT) is known to be effective as a treatment for low mood and anxiety when delivered one to one by an expert practitioner, however this can be expensive and many services have waiting lists and delayed access. A range of additional ways of increasing access to services includes the offer of online courses such as computerised CBT as a possible additional pathway for care. This project aims to test the feasibility of a pilot randomised controlled trial of an online CBT-based life skills course with Chinese-speaking international students experiencing low mood and anxiety.

**Methods/design:**

Chinese-speaking international students with symptoms of low mood and/or anxiety will be recruited from the University of Glasgow, Scotland. Participants will be remotely randomised to receive either immediate access (IA) or delayed access (DA) to a guided/supported online CBT-based life skills package, the “Living Life” package (Chinese version). Participants will be randomly assigned to IA or DA to the intervention. The primary end point will be at 3 months when the delayed group will be offered the intervention. Levels of depression, anxiety, social functioning and satisfaction will be assessed.

**Discussion:**

This pilot study will test the trial design, ability to recruit, gather completed questionnaires, test drop-out rates and investigate completion and acceptability of the package. The study aims to reduce uncertainties about the delivery of a future substantive study and will also inform a sample size calculation for that subsequent substantive randomised controlled trial (RCT) which will be carried out to determine the effectiveness of the online package in improving low mood and anxiety in the Chinese-speaking student population.

**Trial registration:**

Current Controlled Trials ISRCTN30816908

## Background

An increasing number of international students who speak Chinese choose to study overseas, including in Scotland. These students face many challenges such as adjusting to the foreign educational, social, economic, interpersonal and political environment. They can also experience significant fresh challenges to world views such as different perceptions of democracy, freedom, openness, equality, liberty and individualism.

International university students experience a high degree of stress and distress and encounter various types of challenges and problems [1–3]. According to findings from the Mental Health Foundation, nearly one-fifth of adults living in the UK experience anxiety or depression. Depression amongst international students has been found to be up to 60 % higher than in host nation students [4]. Problems such as symptoms of depression are common and can affect students’ development and growth during their time studying overseas. Treatments for depression and anxiety include antidepressants and talking therapies. NICE [5] reviewed the evidence base for talking therapies and recommends the use of self-help resources based on a cognitive behavioural therapy (CBT) model for low mood, anxiety and depression. However, access to evidence-based psychological/talking therapies can be difficult, due to a significantly larger demand than supply and also the reluctance by some to access help for mental health difficulties [5]. Problems with accessing services and a scarcity and inequitable geographic distribution of accredited therapists all contribute to long waiting times for therapy [6]. In addition, international students also have little access to CBT in their native language and which takes into account their background and culture. More widely in the NHS, NICE recommends that access to CBT can also be provided using so-called low-intensity approaches such as CBT-based books and also delivery via computerised CBT (cCBT). CBT resources are recommended for the treatment of depression and anxiety in patients who have mild to moderate mental health problems.

The effectiveness of cCBT resources for use in Chinese-speaking populations has only been investigated to a limited extent. Choi et al. [7] supported the efficacy and acceptability of a computerised cognitive behavioural therapy (cCBT) program in reducing symptoms of depression in Chinese Australian adults (not students). In this study, the Chinese depression cCBT program, “Brighten Your Mood”, consisting of an 8-week program with six CBT online educational lessons, homework assignments, Chinese or English additional resources and weekly telephone support, was adopted to treat Chinese Australians with depression. Fifty-five Chinese Australians experiencing depression were randomly allocated to either an immediate treatment group or to a delayed access control group. After treatment, immediate access group participants reported significantly reduced symptoms of depression on the Chinese versions of the Beck Depression Inventory (CBDI) and Patient Health Questionnaire-9 item (CB-PHQ-9); when compared to the control group. Participants also rated the online treatment procedure as acceptable, and improvements were maintained at the 3-month follow-up. Furthermore, Kwok et al. [8] carried out a study investigating the effectiveness of an online CBT package for family caregivers of people with dementia. Thirty-six carers participated in a 9-week online CBT course. Based on the pre- and post-test on the Chinese version of the Neuropsychiatric Inventory Questionnaire and two domains of the Revised Scale for Care-giving Self-Efficacy, a statistically significant reduction in family caregivers’ distress was evident following the intervention.

Previous work on the need to culturally adapt CBT treatments for Chinese-speaking populations has been done. Ying et al. [9] explored how depression is conceptualised by Chinese American college students and considered the differences between Chinese and American perspectives on illness. Whereas American culture takes a dualist approach, where mind and body are viewed as separate entities; the mind, body and the social context are all integrated with each other within the Chinese medical model. They found that students with greater experience of living in a western culture seem to start to conceptualise depression in a very similar way as the host nation. They attributed this to the increased cross-cultural contact experienced by the student group.

However, no studies to date have used computerised CBT that has been culturally adapted for Chinese-speaking international students. Such packages might provide an effective intervention for students facing low mood and anxiety. Gellatly et al. [10] have suggested that the offer of such resources is more effective when guidance/support is provided.

### The student life skills training intervention

The study will test the delivery of an educational life skills package, “Living Life (LL)–Chinese version”, with the option of Mandarin and Cantonese Chinese language resources. The course teaches key life skills and is based on an existing CBT model with a strong educational focus. The content has been evaluated as a series of face-to-face classes [11, 12]. The free-access English language website is widely used with just under 30 million hits a year. The pilot trial aims to clarify many areas of uncertainty of the Chinese version of the Living Life course that would need to be addressed before moving to a future larger substantive study; for example, the ability to—recruit Chinese-speaking international students, deliver the Chinese version course and support the course and gather baseline and follow-up data of anxiety and depressive symptoms.

### Aims

The aim of this study is to recruit Chinese-speaking international students based in the West of Scotland and experiencing symptoms of depression and/or anxiety. We will include both those diagnosed with depression as well as those with raised mood scores without a formal diagnosis.

In this pilot study, the main aim will be to investigate take-up, drop-out and completion rates of the online course and the completion rates for data collection.

Secondary outcomes will be mood ratings at 3 months. We will use changes in the Patient Health Questionnaire 9 (PHQ-9) [13] score to provide data relating to the effect of the intervention on depression levels. The PHQ-9 is our chosen primary outcome measure for the future substantive RCT and provides a valid and reliable measure of depression. It will provide an indication of efficacy and, together with the drop-out/retention rate, will be used to provide a power calculation for the future larger RCT; providing evidence that a change in depression levels between the intervention and control groups can be observed. This is an unfunded PhD study to establish viability of applying for a substantive later funded RCT.

The specific research questions of the study are outlined below.

### Research questions

Primary question:

- Is the study design feasible—is it possible to recruit Chinese-speaking international students from a University setting, randomise participants and collect data at baseline, 3 months and 6 months?

Secondary questions

b. To what extent will participants adhere to the online intervention?
c. Is the Living Life package acceptable to Chinese-speaking students?
d. How many participants will be needed for a sufficiently powered future RCT?

By gathering information about uptake, retention, ability to gather data, as well as obtaining an estimate of treatment effect, it will be possible to complete a power calculation to estimate the sample size required for the future substantive study.

## Methods/design

### Overview

This is a pilot study with a randomised controlled design. This study will investigate the possibility of delivering the culturally adapted Living Life online life skills resource to Chinese-speaking international students. In this RCT, 50 % of participants will be randomly allocated to receive immediate access (IA) to the Living Life (LL) online intervention and 50 % will be in the delayed access control group (DA). The primary end point is 3 months at which point the control arm will be invited to begin the intervention. A further follow-up point will be at 6 months post-randomisation. This 6-month time point was chosen as a comparative, reasonable follow-up length to fit the structure of the academic year.

### Participants

The aim is to recruit up to 50 participants in total to provide an indication of the take-up, use and impact of the course on low mood or anxiety; and identify any problems with recruitment, delivery of the intervention and completion of evaluation measures. Such problems could be recruitment difficulties, drop-out during the intervention, poor adherence to the online course and failure to complete follow-up measures.

Inclusion criteria:

To enter the study, individuals must be Chinese-speaking (Mandarin or Cantonese) international students aged 18 years of age or more, registered as students (undergraduate or postgraduate) at the University of Glasgow and living in the UK for the next 2 months. This is to ensure they are in the UK when using the intervention so that appropriate support can be offered in the event of identified risk. Current symptoms of depression with the score 5+ on the PHQ-9 [13] depression questionnaire and/or a score of 5+ on the generalised anxiety disorder −7 (GAD-7) [14] scale (Chinese language versions) must be present for eligibility. Participants will be required to have broadband web access and also be willing and able to use the online course.

Exclusion criteria:

Participants will be excluded if they do not fulfil the inclusion criteria or are currently receiving specialist mental health treatment or current psychotherapy/counselling. Students who are on antidepressants will not be excluded, but we will record the drug, dose and length of time on the medication.

### Procedure for recruitment

Chinese-speaking international students (undergraduate and postgraduate) will be recruited directly from the community. This will be predominantly through the Glasgow University Counselling and Psychology Service and also using posters, direct emails and adverts; as well as through the University Chinese society.

Individuals who respond to the adverts will be directed to the study recruitment website where they can read the Participant Information Sheet and find out more about the study and how to take part. They can then contact the research team to ask for further information. A consent form will be sent to all potential participants along with an Eligibility Questionnaire and a further copy of the Participant Information Sheet.

The consent form requests permission to use anonymised eligibility data even if a participant is not suitable for the study in order that we can better understand take-up and drop-out and describe basic demographic details of those not taking up the study. The eligibility questionnaire collects demographic information, such as participants’ age, date of birth, current education status, gender, whether they have access to the internet, information regarding previous or ongoing mental health treatment and also whether they have at any point been diagnosed with low mood or anxiety. The eligibility pack also includes the PHQ-9 [13] and GAD-7 [14] in order to assess mood.

### Randomisation

Once eligible participants have completed the consent form, they will then be randomly assigned to one of two groups. Participants’ ID numbers will be passed to a separate researcher who will use the randomisation function in Excel to remotely assign participants to the immediate access (IA) group or the delayed access control (DA) group. As it is a pilot study, we will not stratify for any variables during randomisation. The randomisation will be done remotely by a researcher who will not be involved in the final data analysis. After randomisation, the two groups of participants will be followed up in exactly the same way. The most important advantage of remote, computerised randomisation is that it minimises allocation bias, balancing both known and unknown prognostic factors, in the assignment of treatments.

All participants will then be emailed the necessary information regarding the study for the two different groups of participants. Participants will have the option of contacting the research team to get practical support in accessing the online package. All such contacts will be recorded in a contact log (participant, time/frequency and content of support need).

### Immediate access (IA) group

Participants randomised to IA will be given log in details for the LL online intervention and will begin receiving weekly support via automated emails in either Mandarin or Cantonese. In addition, personalised short weekly support sessions will be offered by email from a Chinese-speaking counsellor. Support will focus on encouraging module completion and practicing what has been learned. Copies of emails will be retained to check adherence to the Plan, Do, Review model [15] of support. This model uses two worksheets (planner and review sheets) to apply what is learned in practice.

### Delayed access control (DA) group

Participants randomly allocated to the delayed access group will be informed that they will be given access to the online course after a delay of 3 months. As in the IA group, all participants will continue with any usual treatment as required.

### Intervention

The “Living Life” Chinese version intervention is an online set of resources including e-books, printable worksheets—together with linked audio modules. The package contains eight modules that teach a range of CBT-based life skills. Modules involve slides with audio and take between 25 and 45 min to complete. Each focuses on a different common problem faced by people when they feel low or anxious.

The eight modules are:

- Session 1: “Why do I feel so bad?”—An introduction to the CBT model and the “vicious cycle” of low mood. This module helps participants to understand why they feel the way they do and how their thoughts, feelings, physical symptoms and behaviour are linked.
- Session 2: “I can’t be bothered doing anything”—This module aims to tackle reduced activity. Users are encouraged to consider the things that they have stopped doing or are prevented from doing as a result of their low mood or anxiety and make a plan to re-establish these activities in order to improve their mood.
- Session 3: “Why does everything always go wrong?” – Skills to help tackle negative thinking are taught in this session. Participants are taught how to label unhelpful thoughts, identify their negative thinking patterns and are given various tools to turn these thoughts around to create more positive ones.
- Session 4: “I’m not good enough”—This session teaches how confidence is developed and the ways in which low self-esteem can impact on mood. Confidence building techniques are taught in this module.
- Session 5: “How to fix almost everything”—A problem-solving module teaching the “Easy 4 Step Plan (E4SP)” which shows participants how to break down problems and tackle small parts of the problem in order to overcome challenges. The session focuses on resolving practical problems that users may be experiencing.
- Session 6: “The things you do that mess you up”—This session addresses unhelpful behaviours that may be worsening mood. Participants are encouraged to recognise problem behaviours and create a plan for reducing them.
- Session 7: “Are you strong enough to keep your temper?”—Participants learn to recognise the things that cause them to feel irritable or angry (their “buttons”) and the physical symptoms they experience when they feel angry. They are then taught techniques for managing their anger and reacting differently to challenging people and situations.
- Session 8: “10 things you can do to feel happier straight away”—The final module teaches key lifestyle choices that can improve mood including healthy eating, exercise and closeness with others.

Users are encouraged to make an individualised plan at the end of each session using a Plan, Do, Review structure [15] in order to apply the techniques learned in the modules. The intervention is designed to be accessible and easily understood. This is achieved by omitting complicated CBT terms and replacing these with simpler language, as seen in the titles of the modules. The language used in the modules includes everyday terms to describe symptoms such as stress, distress and low mood rather than more formal diagnostic terms depressive disorder, depression and anxiety etc. This simple way of communicating CBT used is highly accessible [16] and effective [17, 18].

### Cultural adaptations

Simple/traditional Chinese, Mandarin and Cantonese versions of the materials have been made available. A series of facilitated focus groups identified changes required to culturally adapt content for use with this target population. The main finding from the focus groups was that participants felt that all of the research materials (including advertisements and questionnaires) and the online intervention should be written in the Chinese language. The modules were already in Chinese but all of the menu items and any written information on the home page had to be translated. Additionally, English language versions of the modules were removed from the website.

Opinions relating to preferred support were also collected during the focus groups. The majority of participants in the focus groups stated that support sessions delivered via email would be acceptable and in most cases preferable to telephone support. Therefore, the support sessions will be delivered by a support worker based at the student counselling service via weekly emails to participants. These emails will be written in Chinese, as this was expressed as important by participants.

Finally, the layout of the website was adapted following comments from the focus group participants. The full results of the focus group study will be published separately.

### Support

Participants will have the option to sign up to receive weekly automated support emails with the option of traditional or simple Chinese script. As recommended by the focus group work, participants will receive a regular personalised support element; delivered by a Chinese-speaking trained counsellor via email. This will test the support offered for use in the later substantive RCT.

The support is protocol driven and focused on encouraging use and application of the intervention. It is designed to encourage an individualised plan to be made at the end of each session using a Plan, Do, Review structure, with a focus on making changes in their lives and using the materials to support them in doing this. Therefore, the online intervention uses a structured, guided self-help approach that may be appealing to students.

### Follow-up data collection

The main follow-up point will be at 3 months with an additional follow-up assessment at 6 months (see Table 1). The primary outcome in the future substantive study will be PHQ-9 at 3 months.

**Table 1:** Timing of measures taken during the study

Baseline	3 months	6 months
Demographic data	PHQ-9	PHQ-9
PHQ-9	GAD-7	GAD-7
GAD-7	WSAS	WSAS
WSAS	CSQ-8 (IA group only)	CSQ-8 (DAC group only)

Baseline measures include age, gender, course type (undergraduate/post-graduate), ethnicity, mood, anxiety, social function, (Chinese versions where available), antidepressant medication and current psychiatric treatment.

The following measures will be completed online:

#### Patient health questionnaire-9 [13]

PHQ-9 is an open access mood rating questionnaire consisting of nine questions mirroring DSM-IV depression diagnostic criteria and each rated 0–3 giving a maximum score of 27. The use of PHQ-9 on Chinese general hospital outpatients showed a Cronbach’s alpha coefficient of 0.857, suggesting the good internal consistency [19]. Cut-off scores are used to label depression severity as 0–4: minimal depression; 5–9: mild depression; 10–14: moderate depression; 15–19: moderately severe depression; and 20–27: severe depression.

#### Generalised anxiety disorder 7 [14]

The GAD-7 is a seven item questionnaire focusing on symptoms of anxiety. Each item is rated according to the frequency of the described problem in the past 2 weeks. The responses are scored as follows: 0 = “not at all”, 1 = “several days”, 2 = “more than half the days”, 3 = nearly every day’ with a maximum score of 21. The use of the GAD-7 on Chinese general hospital outpatients found a Cronbach’s alpha coefficient of 0.898 and the test-retest reliability was 0.856, indicating good reliability and validity [20]. Scores of 0–5 on this measure indicate mild anxiety, 6–10 = moderate anxiety, 11–15 = moderately severe anxiety and 15–21 = severe anxiety.

#### Work and social adjustment scale [21]

The Work and Social Adjustment Scale (WSAS) assesses social functioning. The WSAS is a five-point questionnaire and addresses issues relating to the individual’s everyday life and functioning and how their mood disorder is affecting these areas. Responses are given on a scale of 0–8, with higher scores indicating higher level of impairment. Those scoring over 20 are likely to have significant problems in their social functioning. The Cronbach’s alpha coefficient of the Chinese version of WSAS with Chinese college students was 0.849 [22].

#### The client satisfaction questionnaire (CSQ-8) [23]

The English language version of the CSQ-8 will be administered post intervention as a measure of satisfaction. The CSQ-8 is an eight-item questionnaire, rated using a four-point Likert scale. Scores range from 8–32, with higher scores indicating greater satisfaction with the intervention in question. The internal consistency of the CSQ-8, measured by Cronbach’s alpha coefficient, ranged from 0.83 to 0.93 in English-speaking populations [23].

In addition, a check of contamination (use of the website by the DAC), use of other online/book resources and any adverse consequences of using the site in the IA arm will be assessed at 3 months.

### Statistical analyses

Analysis will be descriptive statistics, *t*-tests comparing means, chi square analysis of frequency data and ANOVA analyses. Evaluable data and intention to treat analysis will occur. Results will be analysed and interpreted using SPSS. The majority of the results will be simple descriptive data summarising take-up, completion and drop-out rates. Finally, the PHQ-9 data will be used to perform a power calculation for the future large RCT.

## Discussion

### Importance of the project

We have recently completed a systematic review of the use of low-intensity CBT interventions (online or book-based) with Chinese-speaking populations (manuscript in preparation). This found no previous evaluations of such cCBT treatments aimed at Chinese-speaking international students. Liu’s [3] study of mental health problems in Chinese international college students found that depression is significant in this group, and that access to help in the appropriate language can be limited within English-based student counselling and NHS services. The current pilot study will help inform whether an online low-intensity CBT approach that would increase access to treatment can be evaluated in this population and help define the sample size required to answer the question of clinical effectiveness.

### Knowledge added to the literature

In the pilot study, the sample sizes are chosen to provide the information required. They are deliberately not informed by a power calculation as the purpose of this study is to provide the information required to test delivery of the components of the study and to provide a power calculation for a future substantive study. Moreover, the primary outcome will be the take-up, drop-out and completion rates of the online course and the collection rates for data. Secondary outcomes will be mood rating scales using descriptive statistics. We will use changes in PHQ-9 score to provide an impact of effect of the intervention on mood at 3 months, which will be the primary outcome for the future substantive RCT.

In addition, the results of the current study relating to changes in PHQ-9 and GAD-7 scores could inform and guide practitioners about the mental health needs of Chinese international students and the difficulties faced when they study overseas [3].

### Benefits to participants

Participation within this research will allow Chinese-speaking international students who are experiencing symptoms of anxiety and depression to take part in an online CBT-based course for stress and low mood in their own language. There is evidence supporting the use of the CBT self-help approaches for depression and anxiety by teaching problem-solving skills, assertiveness, tackling negative thinking and increasing activity levels [11, 12, 24, 25]. By using the package, it is hoped that participants may learn new skills to help with symptoms of low mood, anxiety or depression. The satisfaction data will help us to gain a key insight into how online websites can be best delivered and supported in this setting.

Little previous research has examined the presence of adverse impacts of using cCBT. We are therefore monitoring progress using support contacts and in addition have added a question at follow-up to examine adverse consequences in the IA arm. It is unlikely that participants’ condition will worsen significantly during the study. We do however have risk management protocols in place if needed, as outlined in the following section.

### Ethical considerations

There are a number of ethical considerations that have been taken into account in the preparation of this protocol. These are as follows:

#### Recruitment

Recruitment into the study will involve self-referral via web links from established websites, through a project-specific recruitment site, via email, posters and via advertisements. This will involve liaison and support from the University of Glasgow Counselling service, and the University Chinese Society. No individuals will be recruited from NHS sites.

#### Consent

We will be recruiting only participants who are aged 18 years or over. Informed consent will be obtained from all participants prior to them entering the study. Participants will have the opportunity to ask questions about the study before being asked to complete the consent form. Participants will only be given access to the intervention once eligibility has been established and informed consent given.

#### Risk management

The Participant Information Sheet will give suggestions for sources of additional/urgent support including their GP, NHS24, student counselling, A + E and Samaritans. Additionally, the study website contains a tab detailing sources of urgent help. Finally, all participants will be assigned a Chinese-speaking support worker based at the University Student Counselling who will contact them each week to monitor their progress. Therefore, participants will have the opportunity to discuss any deterioration in their mood, in which case the support worker would inform the clinical lead of the study, CW.

#### Confidentiality

Any personal information or data collected will be kept in a locked filing cabinet in the researchers secure office or in password-protected files or memory sticks which only the researchers will have access to. Data sets used for analysis will hold only participant ID numbers, not personally identifying information. Personally identifying information will be linked to a study ID number and details kept in a separate password-protected Excel file.

### Dissemination

We aim to disseminate the findings of the study in an open access journal publication and via conference presentations. The journal publication will present the study findings with the data being used to inform the future RCT protocol and a grant application. A short study outcome sheet will be sent to all participants who have taken part, and the findings will be posted on the study website. We will also disseminate findings via newsletters on the www.livinglifetothefull.com website, which receives around 30 million hits a year from members of the public and practitioners.

## Trial status

Ethical approval has been granted by the College of Medical, Veterinary and Life Sciences Ethics Committee for Non Clinical Research Involving Human Subjects, University of Glasgow, Ref. 200120022. The website has been designed and tested. Recruitment is expected to begin in October 2014. Final outcome data will be collected in May/June 2015.

## Abbreviations

CBT: cognitive behavioural therapy
cCBT: computerised cognitive behavioural therapy
DAC: delayed access control
IA: immediate access
RCT: randomised controlled trial
